# Antifouling Polysulfone/Multi-Walled Carbon Nanotube/Terbium Oxide Nanocomposite Nanofiltration Membrane for Dye Removal Applications

**DOI:** 10.3390/polym18101165

**Published:** 2026-05-09

**Authors:** Abeer M. Alosaimi

**Affiliations:** Department of Chemistry, College of Science, Taif University, P.O. Box 11099, Taif 21944, Saudi Arabia; a.alosaimi@tu.edu.sa

**Keywords:** nanocomposite, nanofiltration membrane, polysulfone, carbon nanotube, antifouling, dye removal, terbium oxide

## Abstract

Polysulfone (PSF) nanofiltration membranes incorporating oxidized multi-walled carbon nanotubes (o–MWCNTs) and terbium oxide (Tb_2_O_3_) nanoparticles were fabricated via the non-solvent-induced phase inversion technique. The effect of Tb_2_O_3_ loading (0, 1, 3, and 5% *w*/*w*) on membrane morphology, hydrophilicity, water permeability, dye rejection, and antibiofouling performance was systematically investigated. Membrane structure was characterized by FTIR spectroscopy, SEM, EDX, XRD, and water contact angle measurements. The results confirmed the successful incorporation of Tb_2_O_3_ within the membrane matrix, and morphological analysis revealed a relatively dense membrane structure without macrovoid formation. Filtration experiments conducted in a dead-end cell under pressures of 1–4 bar demonstrated a maximum water flux of 53 L m^−2^ h^−1^, with dye rejection exceeding 99.9% for both methylene blue (MB) and Congo red (CR) at 4 bar. Antibiofouling performance, evaluated by colony-forming unit analysis, revealed bacterial growth reductions of 59% against Gram-negative *Escherichia coli* and 89% against Gram-positive *Candida albicans*, attributed to the dark-active generation of reactive oxygen species by Tb_2_O_3_, eliminating the need for UV irradiation. These results demonstrate that the synergistic integration of o–MWCNTs and Tb_2_O_3_ effectively addresses the permeability-selectivity trade-off and mitigates biofouling limitations associated with pristine PSF membranes, thereby offering a promising multifunctional platform for sustainable industrial wastewater treatment.

## 1. Introduction

The rapid industrialization and expansion of textile, leather, paper, and dye manufacturing industries have led to extensive discharge of colored effluents into aquatic environments. Synthetic dyes are considered one of the most problematic pollutants in wastewater because of their complex aromatic structures, high stability, and resistance to biodegradation. Even small concentrations of dyes can significantly reduce light penetration in water bodies, thereby affecting photosynthetic activity and aquatic ecosystems. In addition, many dyes and their degradation products exhibit toxic, mutagenic, and carcinogenic effects, posing risks to both human health and the environment [1]. Consequently, development of efficient technologies for dye removal from wastewater has become an important research priority in environmental engineering and membrane science [2,3].

The main techniques used for dye removal from wastewater are adsorption, coagulation, flocculation, advanced oxidation processes, and biological treatments [4,5,6,7,8,9]. However, these methods often suffer from some limitations, such as high operational costs, production of secondary pollutants, and low efficiency for stable dye molecules. Membrane separation techniques are considered promising alternatives for dye removal due to their high efficiency, operational simplicity, low energy consumption, and ability to remove a wide range of pollutants without the need for chemical addition. Membrane-based processes—such as ultrafiltration, nanofiltration, and reverse osmosis—have shown significant potential for treating dye-contaminated wastewater. However, several challenges still hinder their large-scale implementation, for example, membrane fouling, which results from the accumulation of dyes, organic matter, and colloidal particles on the membrane surface, leading to flux decline and increased operational costs due to frequent cleaning or membrane replacement. In addition, concentration polarization near the membrane surface can reduce separation efficiency and further accelerate fouling.

Filtration membranes are made from different polymer materials such as cellulose acetate, polyacrylonitrile, polyvinyl chloride, polysulfone, polyether sulfone, and polyamide [10]. Among these, polysulfone (PSF) has attained considerable attention due to its excellent thermal stability, chemical resistance, and enhanced mechanical performance, making PSF a promising candidate for formulating ultrafiltration and nanofiltration membranes for wastewater treatment applications. Moreover, PSF membranes exhibit good resistance to oxidative and hydrolytic degradation, enabling their use under harsh operating conditions [11]. However, native PSF membranes are relatively hydrophobic, which can lead to membrane fouling and reduced permeability during filtration processes. Therefore, various modification approaches have been developed to enhance the hydrophilicity, permeability, and antifouling performance of PSF membranes [12]. Membrane modification approaches include surface and bulk grafting, coating, blending, and formation of nanocomposite membranes through the incorporation of nanoparticles [13].

One effective strategy for improving membrane properties is the incorporation of inorganic nanomaterials or metal oxides into the polymer matrix to produce mixed matrix or nanocomposite membranes. The addition of nanoparticles can significantly enhance membrane surface properties, pore structure, and separation performance. The incorporation of oxidized carbon nanotube (CNT) and graphene oxide largely enhances membrane hydrophilicity, increases water flux, improves pollutant removal efficiency, and imparts antimicrobial activity [14,15]. The incorporation of TiO_2_, ZnO, and metal–organic frameworks into PSF membranes has been reported to enhance water flux, dye rejection, and antifouling performance [16,17,18,19]. These additives enhance membrane hydrophilicity, impart additional functional properties such as antimicrobial activity, and promote photocatalytic degradation of organic contaminants, thereby improving the overall efficiency of wastewater treatment systems. However, they also have several important drawbacks. One major issue is nanoparticle agglomeration, where effectiveness is reduced and defects are created. Poor interfacial compatibility between the polymer matrix and nanomaterials can lead to weak adhesion, resulting in reduced mechanical stability and possible leaching of nanoparticles. Another concern is membrane fouling, as some nanomaterials may increase surface roughness and promote the accumulation of contaminants [20].

Rare-earth metal oxides have recently gained increasing attention in environmental remediation due to their unique electronic structure, high chemical stability, and catalytic properties [21]. Among them, terbium oxide (Tb_2_O_3_) exhibits promising physicochemical properties such as high surface activity, good thermal stability, and a strong affinity toward organic molecules [22]. The incorporation of Tb_2_O_3_ nanoparticles into polymeric membranes can potentially enhance adsorption capacity and catalytic activity toward dye molecules while simultaneously improving membrane hydrophilicity and structural stability, making Tb_2_O_3_ a promising additive for the fabrication of advanced composite membranes for wastewater treatment applications.

Membrane fabrication techniques are used to control pore size, morphology, and overall structure for specific separation applications. Common methods include phase inversion, where a polymer solution forms a porous membrane via solvent–nonsolvent exchange; electrospinning, which produces high–surface area nanofibrous membranes; interfacial polymerization, used to create thin selective layers for nanofiltration and reverse osmosis; as well as melt mixing and in situ polymerization. Additional approaches such as layer-by-layer assembly and sol–gel processing offer precise control over film structure and composition. By adjusting these techniques, researchers can tailor membrane properties like permeability, selectivity, and mechanical strength for targeted uses [23,24,25].

Therefore, preparation of PSF/CNT /Tb_2_O_3_ composite membranes represents a promising approach for improving membrane performance in dye removal from wastewater. By combining the robust PSF structural properties with the CNT hydrophilic and capillary performance and the Tb_2_O_3_ nanoparticle functional advantages, such composite membranes are expected to exhibit enhanced permeability, improved dye rejection, and better resistance to fouling. The preparation, characterization, and evaluation of these nanocomposite membranes for dye removal will be investigated. This work provides the first systematic structure–property–performance map for this novel ternary hybrid system, highlighting its potential as a multifunctional membrane platform for sustainable wastewater treatment.

## 2. Materials and Methods

### 2.1. Chemicals and Reagents

Polysulfone pellets (MW = 60,000 g/mol) were purchased from Acros Organics Co. (Geel, Belgium). Multiwall carbon nanotubes (MWCNTs, purity ≥95%, outer diameter 10–20 nm, length 10–30 μm) and Tb_2_O_3_ is powder, a purity of ≥99.9%, was purchased from Nanotechnology Co. Ltd. (Cairo, Egypt). Congo red and methylene blue were procured from Alfa Aesar (Ward Hill, MA, USA). All other reagents and solvents were purchased from Sigma-Aldrich (St. Louis, MO, USA).

### 2.2. Functionalization of MWCNTs

Oxidation of MWCNTs was performed through a thermal oxidation experiment to incorporate oxygen-containing functional groups, such as –OH and –COOH, on their surfaces. This process was carried out in a muffle furnace under an air atmosphere; 1 g of MWCNT was placed in a ceramic crucible and heated to 300 C for 2 h. The sample was then allowed to cool naturally to room temperature inside the furnace to prevent moisture uptake. To increase the oxidation efficiency, 0.5 g of the oxidized MWCNT product (o–MWCNTs) was dispersed in 100 mL of H_2_O_2_ and sonicated in an ultrasonic bath for 30 min. After reaction, the suspension was filtered through a 0.45 µm Millipore nylon filter membrane. The resulting product was washed several times with distilled water until a neutral pH was achieved and was subsequently dried at 80 °C in a vacuum oven for 24 h.

### 2.3. Preparation of Nanocomposite Nanofiltration Membrane

The nanocomposite nanofiltration membranes were prepared using the phase inversion technique as described in previous studies [26,27]. Briefly, CNT was suspended in dimethylformamide (DMF) solvent for approximately 2 h in a sonicator water bath, followed by the addition of Tb_2_O_3_. The mixture was sonicated again for 1 h to ensure uniform dispersion. PSF was subsequently added to the dispersed mixture to obtain a polymer solution with 18% solid content. The concentration of o–MWCNT was fixed at 5% *w*/*w*, whereas Tb_2_O_3_ was incorporated at varying concentrations of 1, 3, and 5% *w*/*w* relative to PSF. After complete dissolution, the polymer solution was allowed to stand for approximately 6 h to eliminate entrapped air bubbles. The resulting polymer mixture was cast on a clean, dry glass plate using a casting knife with a thickness of 250 µm. The cast film was immediately immersed in a coagulation water bath and left overnight to ensure complete phase inversion and formation of the nanocomposite membrane. The composition of the prepared membrane is summarized in Table 1.

### 2.4. Characterization of the Prepared Membrane

The chemical structure and morphological properties of the obtained nanocomposite membranes were characterized using various instrumental techniques. Reactive functional groups were analyzed using attenuated total reflectance Fourier transform infrared spectroscopy (ATR–FTIR; Jasco FTIR-430, Tokyo, Japan) over a spectral range of 400–4000 cm^−1^. Surface and cross-section topography were examined using field emission scanning electron microscopy (SEM; Quanta FEG250, FEI, Hillsboro, OR, USA). Prior to analysis, dried membranes were sputter-coated with gold using an S150A sputter coater (Edwards, Crawley, UK). Energy dispersive X-ray (EDX) spectroscopy, coupled with an SEM system, was used to determine the elemental composition of the composite membrane. The crystallinity of the membrane was evaluated using X-ray diffraction (SEIFERT XRD 3003 TT diffractometer, Ahrensburg, Germany) equipped with a primary monochromator (CuK radiation, λ = 1.5406 Å, 2θ = 5–80°). The hydrophilicity of the composite membrane was assessed through contact angle measurements. A droplet of pure water (10 µL) was gently placed on the membrane surface at room temperature, and the contact angle between the membrane–water and water–air interfaces was measured using a camera connected to a computer.

### 2.5. Evaluation of Nanofiltration Membrane Performance in Dye Removal

The water flux and dye rejection efficiency of the prepared nanocomposite membrane were evaluated using a dead-end filtration cell with an effective membrane area of 14.6 cm^2^. Dye solutions of varying concentrations were filtered under applied pressure ranging from 1 to 4 bar at 25 °C. The concentrations of dyes before and after filtration were determined through UV–Vis spectroscopy at wavelengths of 494 nm and 668 nm for Congo red and methylene blue, respectively.

The water flux of the membrane (J, L/m^2^·h) was calculated using the following Equation (1):

(1) $$J=\;\frac{V}{A\;\Delta t},$$

where V is the permeate volume (L), A is the effective membrane area (m^2^), and ∆t is the filtration time (h). The dye rejection (R, %) was calculated using Equation (2):

(2) $$R\;(\%)\;=\left(1\;-\;\frac{C_{\;\mathrm{permeate}}}{C_{\;\mathrm{feed}}}\;\right)\;\times \;100,$$

where C_permeate_ and C_feed_ represent dye concentrations in the permeate and feed solutions, respectively.

All measurements were performed in triplicate, and the data are presented as mean ± standard deviation. The deviation values ranged from ±0.1 to ±01.1, reflecting good reproducibility of the experimental system.

### 2.6. Antibacterial Performance of the Membrane

The antibacterial performance of the composite membrane was assessed using the colony-forming unit (CFU) method.

The antimicrobial activity of different composite membranes was evaluated by determining the percentage reduction in CFU against the Gram-negative bacterium Escherichia coli, the Gram-positive bacterium Staphylococcus aureus, and the pathogenic fungus Candida auris using the shake flask method. Briefly, 20 mL of nutrient broth in small conical flasks was inoculated with 25 µL of bacterial suspension, standardized to 0.5 McFarland (1.5 × 10^8^ CFU/mL). Subsequently, 100 µL of the tested sample was added to the respective inoculated flasks and incubated at 37 °C for 24 h under shaking conditions (120 rpm) [28]. After incubation, serial dilutions (up to 10^−4^) were prepared for each flask containing a sample–culture mixture and corresponding controls for each strain. Aliquots of 100 µL from the diluted suspensions (up to 10^−4^) were spread onto Petri dishes containing solidified nutrient agar and incubated for colony formation. The antimicrobial efficacy was determined by calculating the percentage reduction in growth rate (R, %) for treated samples relative to the control (untreated) using the following Equation (3):

Relative reduction (%) = (A − B/A) × 100,(3)

where A represents the CFU/mL in the untreated control (pathogenic strains without any treatment), and B represents the CFU/mL in the treated sample.

## 3. Results and Discussion

### 3.1. Characterization of Nanocomposite Membranes

#### 3.1.1. ATR-FTIR

**Figure 1:**
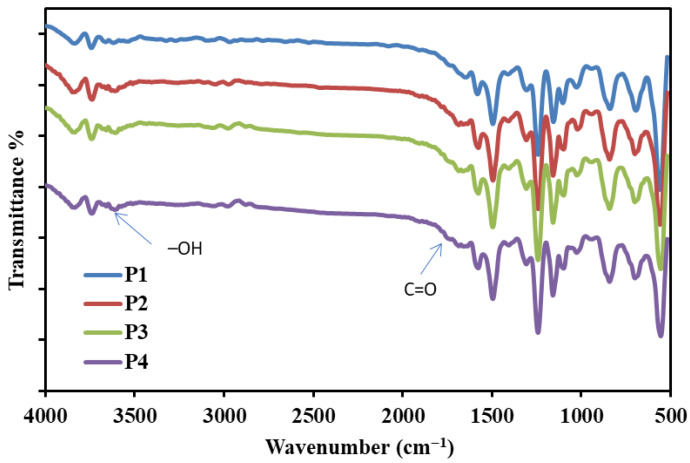
FTIR of the nanocomposite membranes.

Reactive functional groups of the nanocomposite membrane were assessed using ATR-FTIR, as shown in Figure 1. All the prepared membranes exhibited characteristic absorption peaks corresponding to PSF; for example, peaks observed at 1565 and 1488 cm^−1^ represented stretching vibrations of aromatic (C=C) groups, and the peak at 1155 cm^−1^ corresponded to the symmetric stretching of the (O=S=O) group, and that at 1236 cm^−1^ was attributed to the ether (C-O-C) group [29]. Additionally, a peak at 835 cm^−1^ was associated with C-H bending, and peaks at 2969 and 2849 cm^−1^ were attributed to aliphatic stretching vibrations of CH_3_ groups. PSF/o–MWCNT membranes displayed a weak peak at approximately 1704 cm^−1^ corresponding to the stretching vibration of the carbonyl (C=O) group [30]. This peak shifted to 1714 cm^−1^ and exhibited a slight increase in intensity with increasing Tb_2_O_3_ content, suggesting interfacial Lewis acid–base interactions between o–MWCNT carboxylate groups and Tb_2_O_3_ surface sites within the polymer membrane matrix. Furthermore, a peak at 3579 cm^−1^ was attributed to the hydroxyl group (OH) of oxidized CNT.

#### 3.1.2. SEM

**Figure 2:**
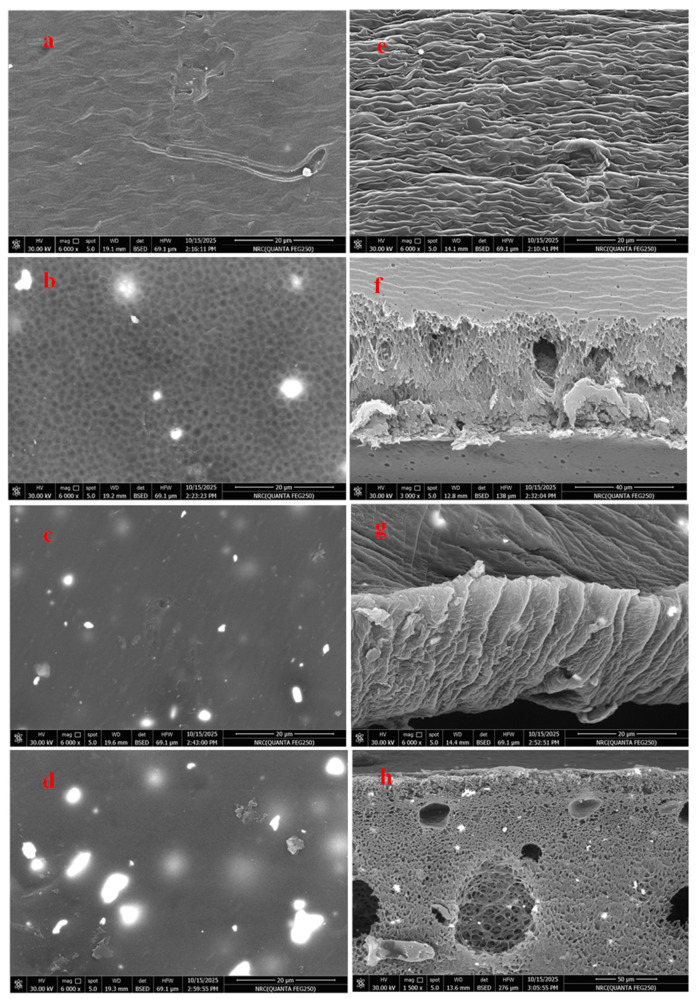
SEM of surface (**left**) and cross-section (**right**) for (**a**,**e**) P1, (**b**,**f**) P2, (**c**,**g**) P3, and (**d**,**h**) P4, respectively.

The surface and cross-section morphologies of the prepared nanocomposite membranes are displayed in Figure 2. As shown, the blank PSF/o–MWCNT membrane surface exhibited a rough, non-porous surface with a nodular structure owing to the hydrophilic nature of CNTs, facilitating their aggregation at the membrane surface to form a nodular structure [31], whereas the membrane cross-section showed an absence of macrovoid formation. Upon incorporation of Tb_2_O_3_, the membrane surface exhibited enhanced porosity (Figure 2b, left), and its cross-section appeared as a spongy structure with the appearance of small macrovoids (Figure 2f, right). Further increases in Tb_2_O_3_ content led to an increase in macrovoid formation in the cross-section, along with an increase in the overall cross-sectional porosity (Figure 2g,h, right). In contrast, the membrane surface showed a dense structure, with no increase in porosity. Although the phase inversion technique employed for membrane fabrication typically induces instantaneous solvent–non-solvent de-mixing, resulting in precipitation of the membrane after immersion in the water coagulation phase and presence of large macrovoids and finger-like structures, which were not observed in the present system [32]. This behavior may be attributed to the presence of hydrophilic oxidized CNTs and Tb_2_O_3_, which enhance hydrogen bonding interactions and increase the viscosity of the casting solution. Consequently, solvent out-diffusion is favored over nonsolvent (water) in-diffusion, resulting in reduced pore size and membrane porosity [33].

**Figure 3:**
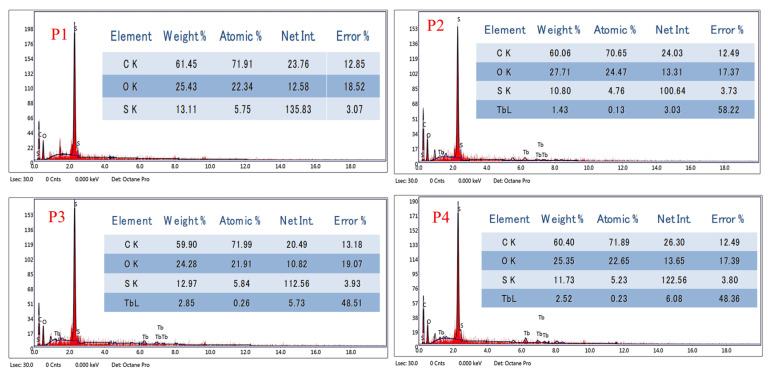
EDX analysis for P1, P2, P3, and P4, respectively.

The elemental structure of the membrane was investigated using EDX coupled with SEM, as shown in Figure 3. The control PSF/CNT membrane showed that the elemental composition of PSF comprised carbon, oxygen, and sulfur with a ratio of 61.45:25.43: 13.11%, consistent with the polysulfone backbone structure. The dispersion of Tb_2_O_3_ within the membrane matrix was confirmed by EDX analysis of Tb at varying contents of 1.43, 2.85, and 2.52% *w*/*w*. Although EDX analysis is not a quantitative technique, it indicates the presence and approximate distribution of this nanofiller within the polymer matrix.

#### 3.1.3. XRD

**Figure 4:**
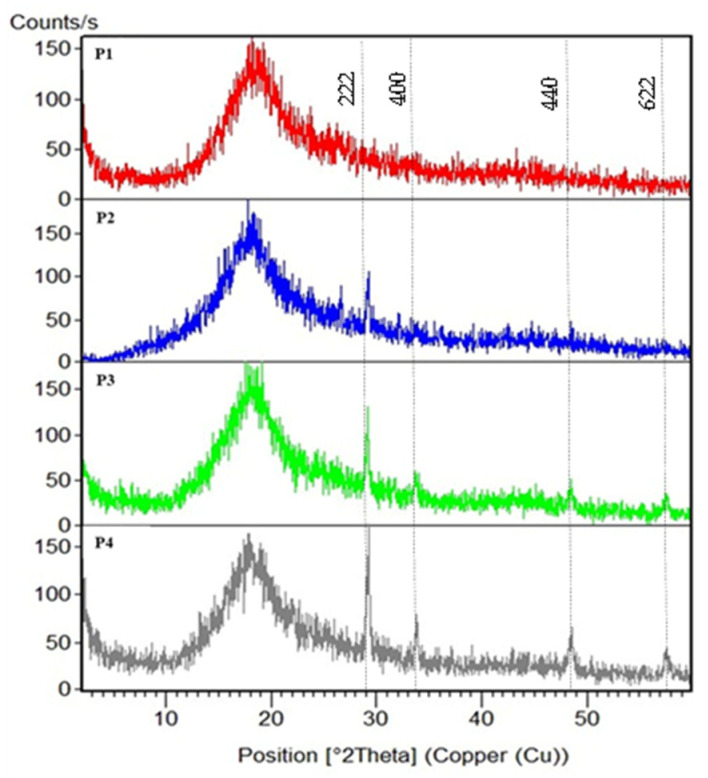
XRD pattern of P1, P2, P3, and P4 respectively.

XRD was performed to assess the crystallinity of the membrane and the dispersion of the inorganic materials within the PSF matrix. Figure 4 displayed the XRD patterns of control PSF/CNT and its nanocomposite with Tb_2_O_3_. The XRD pattern of the control membrane showed a broad peak at about 18°, owing to the amorphous structure of the PSF chains that represents the average inter-planar spacing of the polymer chains [34]. The presence of a sharp diffraction peak at 2theta at about 29° represents the primary strong peak of the (222) plane of Tb_2_O_3,_ with other small peaks at 33.4°, 47.8°, and 56.6° representing (400), (440), and (622) planes of Tb_2_O_3_ [35,36]. The intensity of these well characteristics peaks of Tb_2_O_3_ increased with increasing Tb_2_O_3_ content in the polymer matrix, as shown in the figure.

#### 3.1.4. Contact Angle Measurement

**Figure 5:**
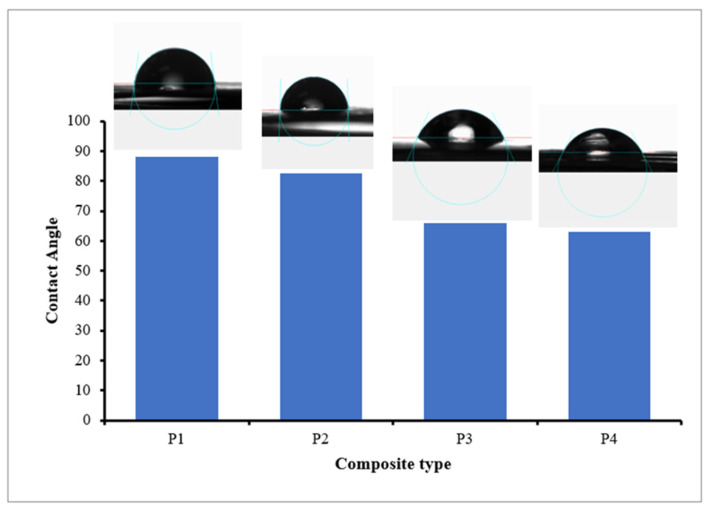
Contact angle measurements of the nanocomposite membranes.

The hydrophilicity of the prepared nanocomposite membranes was assessed using water contact angle measurements. The contact angles of nanocomposite membranes without and with varying concentrations of Tb_2_O_3_ are shown in Figure 5. The contact angle of the control PSF/CNT membrane was 88.2°, indicating its relatively hydrophobic nature [37]. Upon incorporation of Tb_2_O_3_, the contact angle decreased significantly to 82.4, 65.8, and 63° for membranes containing 1, 3, and 5% *w*/*w* Tb_2_O_3_, respectively (Table 2). The increased hydrophilicity, by adding Tb_2_O_3_, may be attributed to the alterations in surface energy and increased porosity of the membrane surface, leading to enhanced wettability and hydrophilicity of the membrane [38,39]. Enhanced hydrophilicity of the filtration membrane is a favorable property in water treatment applications, as it reduces the energy required to drive water through the barrier membrane.

### 3.2. Application of Nanocomposite Nanofiltration Membrane in Dye Removal

**Figure 6:**
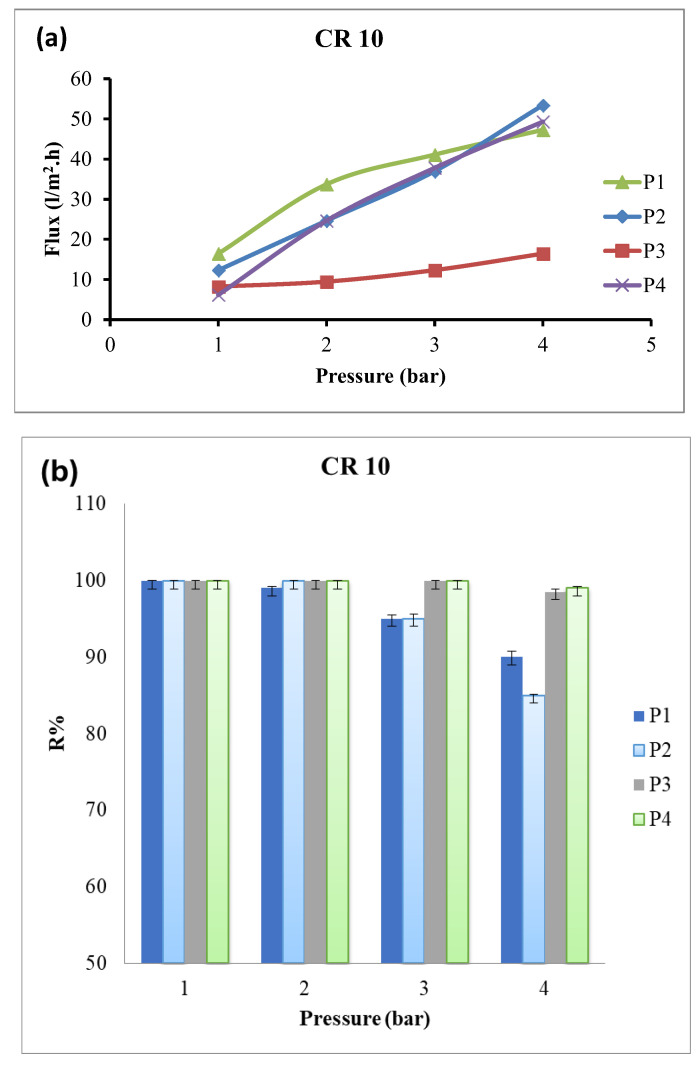
Water flux (**a**) and dye rejection (**b**) of membranes towards Congo red (concentration 10 ppm) at different applied pressures.

Following elucidation, the chemical and physical characteristics of the composite membranes and their performance were assessed for the removal of organic dyes from wastewater. Methylene blue and Congo red were selected as model cationic and anionic dyes, respectively. Filtration parameters, including applied pressure and dye concentration, were assessed using the filtration cell after membrane compaction using distilled water at 4 bar for approximately 4 h or until stable permeability was achieved. Figure 6 and Table 3 indicate the water flux and dye removal efficiency for Congo red (10 ppm) at different applied pressures, ranging from 1 to 4 bar. As expected, water flux increased with increasing applied pressure for all membranes [40], primarily due to improved membrane hydrophilicity. The membrane with the highest Tb_2_O_3_ content (P4) exhibited superior performance, achieving a maximum water flux of 49.32 L/m^2^·h at 4 bar, along with the highest dye removal efficiency at this pressure. The P2 membrane showed the highest water flux of 53.4 L/m^2^·h, likely due to its high porosity, as observed in the SEM analysis; however, its dye rejection decreased to 85%. At low pressures, all nanocomposite membranes demonstrated dye removal exceeding 99%; however, a slight decrease was observed at higher pressures of 3–4 bars, except for the P3 and P4 membranes containing high Tb_2_O_3_ content. Collectively, dye rejection for both cationic and anionic dyes was attributed to the synergistic effect of the novel dual-filler membrane architecture.

**Figure 7:**
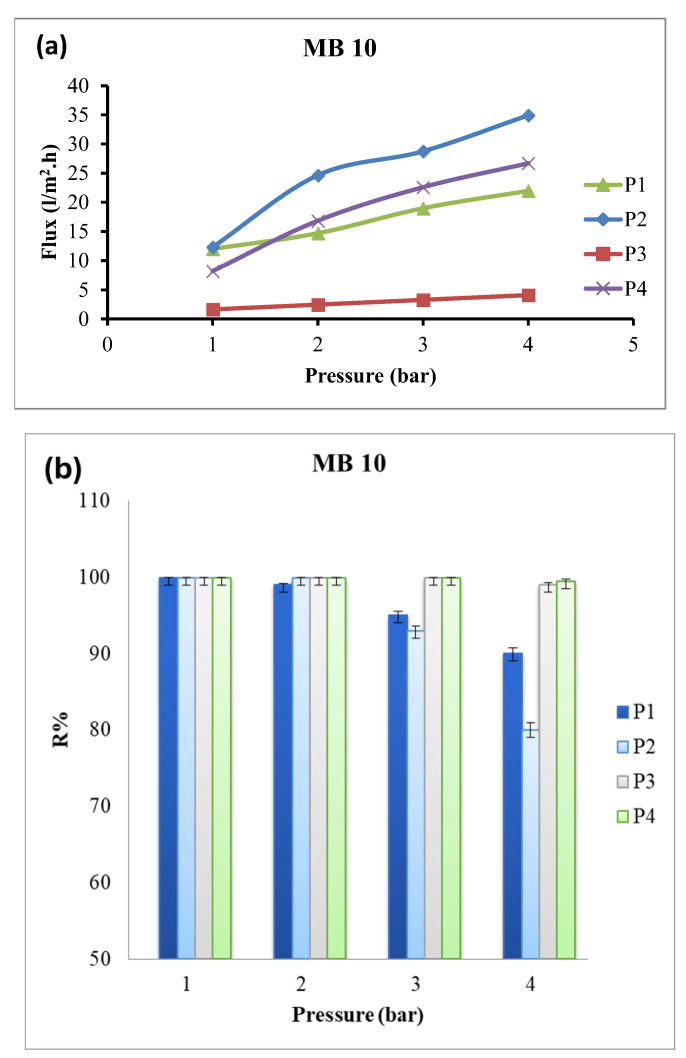
Water flux (**a**) and dye rejection (**b**) of membranes towards methylene blue (concentration 10 ppm) at different applied pressures.

The water flux and methylene blue rejection efficiency of the nanocomposite membranes at applied pressures of 1–4 bar and dye concentration of 10 ppm are presented in Figure 7 and Table 4. The water flux of nanocomposite membranes increased by increasing the applied pressure from 1 bar to 4 bar. Membrane P2 exhibited the highest water flux at all applied pressures, with approximately 34.9 L/m^2^.h at 4 bar, which may be due to its high porosity, as indicated by the SEM analysis. The dye rejection of the membrane decreased with increasing pressure, whereas membrane P4 maintained a stable and high dye rejection efficiency (up to 99.5% at 4 bar). Therefore, membrane P4 was identified as the optimal formulation for further investigation.

**Figure 8:**
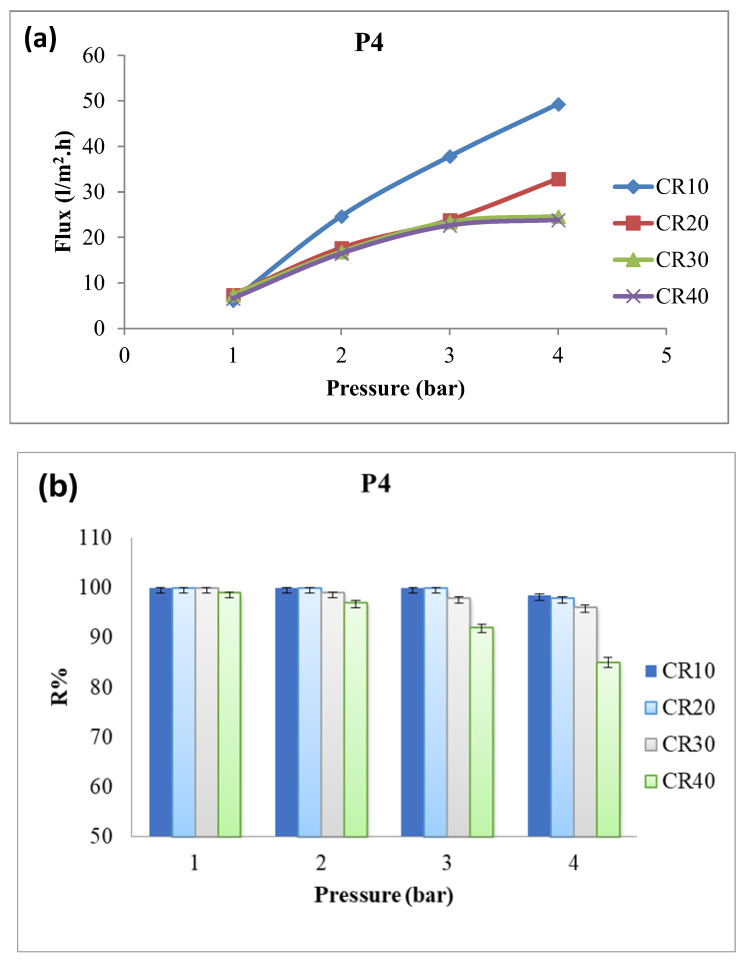
Water flux (**a**) and dye rejection (**b**) of P4 towards different Congo red dye concentrations at different applied pressures.

**Figure 9:**
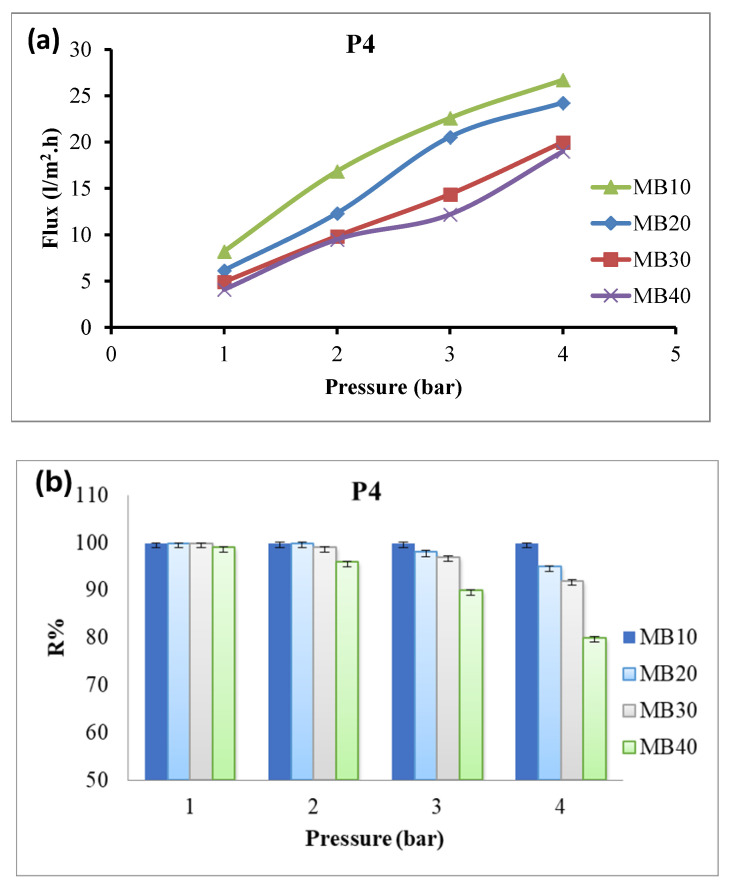
Water flux (**a**) and dye rejection (**b**) of P4 towards different methylene blue dye concentrations at different applied pressures.

The effect of dye concentration (10–40 ppm) on the performance of membrane P4 was investigated at applied pressures of 1–4 bar (Figure 8 and Figure 9 for Congo red and methylene blue dyes, respectively). The water flux of the membrane increased with applied pressure but decreased with increasing dye concentration. This decrease in water flux may be due to increased viscosity of the solution and increased concentration polarization of the dye solution at the membrane surface (Table 5 and Table 6) [41]. Moreover, dye rejection of the nanocomposite membrane decreases with decreasing dye concentration, particularly at higher pressures (4 bar); Figure 8b [42].

Dye rejection performance of the membrane towards different concentrations of methylene blue showed a similar trend as Congo red; however, the decline in rejection efficiency with increasing dye concentration was more pronounced compared with Congo red. This may be due to the lower molecular weight and smaller molecular size of methylene blue (MW = 319.85 g/mol), which facilitates its passage through membrane pores, compared with the higher molecular weight and larger molecular size of Congo red (MW = 696.66 g/mol) [43].

### 3.3. Antibacterial Performance

**Figure 10:**
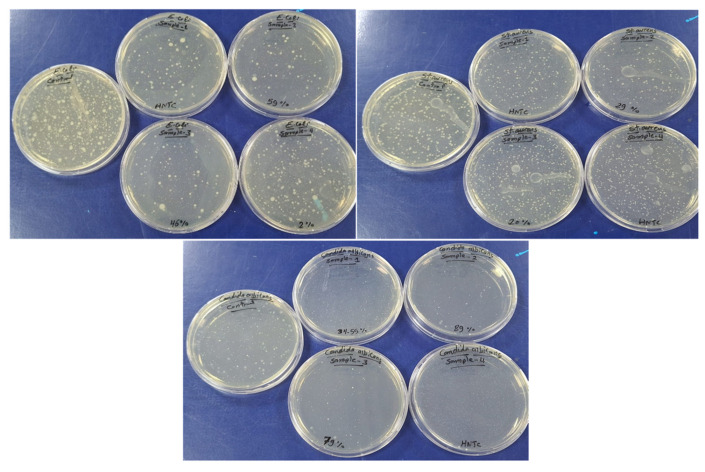
CFU antibacterial activity of the composite membrane towards Gram-positive and Gram-negative bacteria.

The antimicrobial performance of the prepared nanocomposite membranes was assessed using the CFU technique, and the results are presented in Figure 10. The composite membranes exhibited significant antimicrobial activity, with the highest reduction efficiencies observed against both *E. coli* and *S. aureus*, reaching 93.6% and 92.44%, respectively. CFU analysis (Figure 10, Table 7) revealed a clear dose-dependent antimicrobial response with Tb_2_O_3_ loading. The PSF/CNT control exhibited no antimicrobial activity, confirming that the observed effects are primarily attributed to the incorporation of Tb_2_O_3_. Membrane P4 achieved the highest growth reduction of approximately 59% against the Gram-negative *E. coli* and 29% against the Gram-positive *S. aureus*, and approximately 89% against the fungal strain *C. albicans*. The observed antimicrobial activity may be attributed to the dark-active generation of reactive oxygen species, such as ·OH and O_2_^•−^, mediated by the Tb^3+^/Tb^4+^ redox couple on the surface of Tb_2_O_3_ nanoparticles, inducing oxidative damage to microbial cell membranes and disrupting essential metabolic pathways [44,45]. In addition, antimicrobial effects may arise from direct interactions between metal oxide nanoparticles and bacterial/fungal cell walls, leading to the disruption of cellular integrity. The metal oxide nanoparticles may participate in hydrogen bonding and dipolar interactions with various acceptors in the bacterial cell wall, such as amide groups in peptidoglycan [46].

## 4. Conclusions

Novel PSF/o–MWCNT/Tb_2_O_3_ nanocomposite nanofiltration membranes were successfully fabricated via the phase inversion technique with Tb_2_O_3_ loadings ranging from 0 to 5% *w*/*w*. Structural characterization of the obtained composite membranes confirmed uniform dispersion of metal oxide nanoparticles within the polymer matrix, and morphological analysis revealed a macrovoid-suppressed, sponge-like membrane structure with a dense selective top layer across all loading concentrations. Incorporation of Tb_2_O_3_ significantly enhanced membrane hydrophilicity, as evidenced by a reduction in the water contact angle from 88.2° to 63.0° (−28.6%). The optimized PSF/CNT/5-Tb membrane achieved a water flux of 53 L/m^2^·h and dye rejection of ≥99% for both methylene blue (cationic) and Congo red (anionic) at an applied pressure of 4 bar. The antimicrobial activity assessed using CFU analysis demonstrated a 59% reduction against *E. coli*, 29% against *S. aureus*, and an 89% reduction against *C. albicans*, even in the absence of UV irradiation.

## Figures and Tables

**Table 1:** The abbreviations and compositions of the prepared membranes.

Code	O–MWCNTs (%)	Tb_2_O_3_ (%)
P1	5	0
P2	5	1
P3	5	3
P4	5	5

**Table 2:** Water contact angle measurements for all membrane formulations.

Membrane	Contact Angle (°)	Hydrophilicity Change
P1	88.2 ± 1.8	Baseline (hydrophobic)
P2	82.4 ± 1.5	6.6%
P3	65.8 ± 1.3	25.4%
P4	63.0 ± 1.2	28.6%

**Table 3:** Water flux of membranes towards Congo red (concentration 10 ppm) at different applied pressures.

Pressure (Bar)	P1	P2	P3	P4
1	16.44 ± 0.1	12.33 ± 0.3	8.22 ± 0.04	6.165 ± 0.2
2	33.702 ± 0.2	24.66 ± 0.6	9.453 ± 0.06	24.66 ± 0.4
3	41.1 ± 0.4	36.99 ± 0.7	12.33 ± 0.08	37.812 ± 0.5
4	47.265 ± 0.3	53.43 ± 0.8	16.44 ± 0.1	49.32 ± 0.65

**Table 4:** Water flux of membranes towards methylene blue (concentration 10 ppm) at different applied pressures.

Pressure (Bar)	P1	P2	P3	P4
1	12 ± 0.1	12.33 ± 0.15	1.644 ± 0.12	8.22 ± 0.1
2	14.7 ± 0.1	24.66 ± 0.3	2.466 ± 0.24	16.851 ± 0.1
3	19 ± 0.2	28.77 ± 0.5	3.288 ± 0.35	22.605 ± 0.1
4	22 ± 0.3	34.935 ± 0.59	4.11 ± 0.5	26.715 ± 0.2

**Table 5:** Water flux of P4 towards different Congo red dye concentrations at different applied pressures.

Pressure (Bar)	CR10	CR20	CR30	CR40
1	6.165 ± 0.2	7.398 ± 0.1	7.398 ± 0.1	6.576 ± 0.2
2	24.66 ± 0.1	17.673 ± 0.3	16.851 ± 0.1	16.44 ± 0.2
3	37.812 ± 0.3	23.838 ± 0.1	23.427 ± 0.2	22.605 ± 0.1
4	49.32 ± 0.1	32.88 ± 0.2	24.66 ± 0.1	23.838 ± 0.1

**Table 6:** Water flux of P4 towards different methylene blue dye concentrations at different applied pressures.

Pressure (Bar)	MB10	MB20	MB30	MB40
1	8.22 ± 0.02	6.17 ± 0.3	4.93 ± 0.3	4.10 ± 0.3
2	16.85 ± 0.01	12.33 ± 0.2	9.86 ± 0.2	9.50 ± 0.1
3	22.61 ± 0.02	20.55 ± 0.3	14.39 ± 0.4	12.20 ± 0.2
4	26.72 ± 0.04	24.25 ± 0.4	20.00 ± 0.3	19.00 ± 0.3

**Table 7:** CFU growth reduction (%) of PSF/CNT/x-Tb_2_O_3_ nanocomposite membranes.

Membrane	Tb_2_O_3_ (wt.%)	*E. coli* red. (%)	*S. aureus* red. (%)	*C. albicans* red. (%)
P1	0	0	0	0
P2	1	2	0	34.5
P3	3	46	20	79
P4	5	59	29	89

## Data Availability

The original contributions presented in this study are included in the article. Further inquiries can be directed to the corresponding author.
